# Oxidative Stress in Collegiate Cross Country Skiers in Mid- and Post-Seasons

**DOI:** 10.4236/jbm.2024.125009

**Published:** 2024-05-16

**Authors:** Scott P. Jerome, Kriya L. Dunlap, Ava M. Parrish, Matthew L. Lowman, Emily M. Shipman, Lawrence K. Duffy, Arleigh J. Reynolds

**Affiliations:** 1 Department of Chemistry & Biochemistry, University of Alaska Fairbanks, Fairbanks, AK, USA; 2 Institute of Arctic Biology, University of Alaska Fairbanks, Fairbanks, AK, USA; 3 Department of Biology & Wildlife, University of Alaska Fairbanks, Fairbanks, AK, USA; 4 Department of Veterinary Medicine, University of Alaska Fairbanks, Fairbanks, AK, USA

**Keywords:** Oxidative Stress, 4-Hydroxynonenal, Superoxide Dismutase, Athletic Performance, Training Macrocycle

## Abstract

**Purpose:**

The oxidative stress (OS) hypothesis of overtraining syndrome argues that increased production of free radicals through exercise cause muscle fatigue and damage resulting in lower athletic performance. Several studies have investigated OS immediately before and after exercise bouts in a training macrocycle. Our study aimed to compare OS of endurance athletes between a competition macrocycle and the immediate post-season recovery macrocycle. In addition, we aimed to identify athletes who experienced an unexplainable drop in athletic performance during the competition season in order to compare their OS to those who experienced no drop in performance.

**Methods:**

Fifteen members of the University of Alaska Fairbanks cross country ski team volunteered for this study. Blood samples were taken in early February (“mid-season”) and late April (“post-season”). Participants completed questionnaires regarding physical activity and athletic performance at the time of the blood draws. Plasma was analyzed for 4-hydroxynonenal (HNE), nitrotyrosine, nitric oxide (NOX), and superoxide dismutase (SOD). Significance was determined by Wilcoxon and Mann-Whitney tests.

**Results:**

Participants displayed significantly higher (p < 0.05) SOD activity in the post-season (0.02065 U/mL ± 0.006477 SEM) as compared to mid-season (0.04459 U/mL ± 0.005860). Six athletes reported an unexplainable drop in performance (6D). In the post-season, HNE concentration was significantly higher (p < 0.05) for 6D (197.5 μg/mL ± 22.79) than for participants who did not report such a drop (69.80 μg/mL ± 33.59). 6D SOD activity was significantly higher in post-season (0.05048 U/mL ± 0.004688) than mid-season (0.01241 U/mL ± 0.006469).

**Conclusion:**

Signs of oxidative stress and mitigation during the post-season recovery macrocycle were higher in athletes who reported experiencing a drop in athletic performance during the competition season macrocycle.

## 1. Introduction

Of all the diseases, injuries, and illnesses which can negatively affect performance of elite (world class) and sub-elite (collegiate, national, and regional level) endurance athletes, few are more frustrating for athletes, coaches, and physicians than overtraining syndrome (OTS). Characterized by an unexplainable drop in athletic performance, OTS symptoms can also include general malaise, lethargy, disrupted cognitive function, mood swings, depression, and sleep disorders [1]. Meeusen *et al*. describe OTS as a “prolonged maladaptation” of biological, neurochemical, and hormonal regulation systems [2]. The diagnosis of OTS may result when no other explanation for an athlete’s prolonged drop in performance can be identified [1]. A recent search of the PubMed database produced 150 references for “overtraining syndrome”, 73 for “overtraining syndrome diagnosis”, and only two for “overtraining syndrome prediction”. A 2012 Joint Consensus Statement on OTS from the European College of Sport Science and the American College of Sports Medicine identified no convincing evidence as to the causes of the condition [2]. To date, most published studies regarding OTS have focused on prevention, symptoms, diagnoses, and recovery [1]-[7]. A gap in the literature exists with regards to prediction. The basic question, “Who is most susceptible to developing OTS?” remains unanswered.

Several hypotheses exist regarding the etiology of OTS, ranging from chronic glycogen depletion to inflammatory cytokine release [1]. The oxidative stress hypothesis asserts that when oxidative stress becomes pathologic inflammation, muscle fatigue and soreness will result in reduced athletic performance [1]. Tanskanen, Atalay, & Uusitalo found indicators of higher oxidative stress in overtrained athletes than in controls both at rest and during exhaustive exercise, suggesting that oxidative stress plays a role in the pathophysiology of OTS [8]. A study featuring a periodized training protocol for human participants culminated with an overtraining (OT) block resulting in significant increases in three oxidative stress markers (OSMs): isoprostanes, thiobarbituric acid-reactive substances, and protein carbonyls [9]. While studies have revealed a link between OSMs and OTS following acute exercise [8] [9], few if any have examined the relationship between oxidative stress and overtraining over a training or competition macro cycle.

Functional overreaching (FO) is defined as the normal overload, recovery, and super-compensation cycle required for athletic improvement. Nonfunctional overreaching (NFO) shares an overload feature with FO, but an ineffectual recovery period leads to stagnant performance. OT shares an overload period and ineffectual recovery with NFO. However, instead of a performance plateau, performance drops with OT [2]. These three outcomes to athletic training—FO, NFO, and OT—are not completely isolated responses but exist together on a stress/response continuum. This study examines the response to a macro training cycle by examining athletes’ abilities to adapt to stress and reach homeostasis. While it is unclear if any of our participants were overtrained, six of the 15 reported at least one unexplained drop in athletic performance during the competitive season lasting three weeks or longer. Though not conclusive in and of itself, a lasting drop in athletic performance is one diagnostic index for OTS [1]. Our study examined differences in oxidative stress/status markers in athletes who reported at least one unexplained drop in performance during a competition season and those who did not. We sampled participants at two different times of the year: 1) in the middle of the competitive season; and 2) following one month of recovery in the post-season. We hypothesized that those who reported a drop in performance would display significantly greater oxidative stress than those who did not. Furthermore, we hypothesized that those who experienced no drop in performance would show lower oxidative stress in the post-season condition because oxidative stress is associated with muscle fatigue [1]. Analyzing resting OSM values from mid-season and post-season recovery is a comparison few studies have undertaken. These data will add to the body of knowledge of OSMs during periods of exercise and recovery.

## 2. Methods

The study protocol was approved by the Institutional Review Board of the University of Alaska Fairbanks (UAF) (#838437-3). Following a thorough description of the study, including risks and benefits, 15 members of the UAF intercollegiate cross country ski team gave their informed consent to participate prior to data collection. The skiers were training for, and competing in, National Collegiate Athletic Association (NCAA) Division-I regional and national championship events. The group consisted of eight males and seven females (Table 1). All participants had been training for ten to twenty hours per week for no fewer than three months prior to sample collection. The first collection took place in early February, the middle of the competition season (mid-season). The second collection took place in late April, approximately four weeks following the conclusion of the competition season (post-season recovery). All samples were collected at least 15 hours post-exercise.

Participants completed the International Physical Activity Questionnaire (IPAQ) Short Form with instructions from a researcher at both data collection dates. Data from the questionnaires were cleaned and analyzed according to the IPAQ Guidelines for Data Processing and Analysis. A questionnaire to determine hours of athletic training and sport performance history was also administered at mid-season and post-season sampling.

Participants fasted for 12 hours prior to each blood draw, mid- and post-season, with nothing consumed except water. Blood was obtained via venipuncture by a registered nurse trained in phlebotomy. 17.5 mL of blood was drawn per participant into three tubes: 4 mL, 3.5 mL, and 10 mL. The 4 mL and 3.5 mL EDTA tubes were sent to an outside lab (Quest Diagnostics, Madison, NJ) for analysis while samples collected in the 10 mL EDTA tubes were centrifuged, aliquoted into 0.5 mL tubes, and flash-frozen within 30 minutes of sample collection. The resulting serum was stored at −80°C for future analyses.

Plasma samples were analyzed for superoxide dismutase (SOD) (Cayman Chemical, Ann Arbor, MI), nitrotyrosine^†^, 4-hydroxynonenal^†^ (HNE), and nitric oxide^†^ (NOX) (via nitrite and nitrate) (^†^Cell Biolabs, San Diego, CA) according to manufacturer’s instructions.

A power analysis prior to study commencement indicated that a minimum of 12 participants would be necessary for validity. Statistical analysis was performed with Graphpad Prism (version 5, Graphpad Software, Inc.). Normality of data sets was determined using the D’Agostino & Pearson omnibus test. If normal distribution for a particular set was found, data were compared with a paired t-test. If data were not normally distributed, sets were compared with the Wilcoxon matched-pairs signed rank test. Unpaired data were analyzed with the Mann-Whitney test. Outliers were identified with the extreme studentized deviate method (Grubbs’ test). Significant differences are reported at p ≤ 0.05. All error is reported as standard error of the mean (± SEM).

We did not have adequate sample volume from two participants in the mid-season condition for the nitrotyrosine assay. The matched samples from the post-season recovery condition were subsequently not used for data analysis. One data point for SOD activity in the mid-season condition was found to be an outlier. Neither this outlier nor its corresponding value from the post-season recovery condition were used in SOD analyses.

## 3. Results

The IPAQ data indicate that the participants were significantly more active in mid-season than during post-season recovery (Table 1). Six reported experiencing at least one period of unexplainable, reduced athletic performance lasting at least three consecutive weeks during the season. The remaining nine reported no such drop in performance.

**Figure 1.**
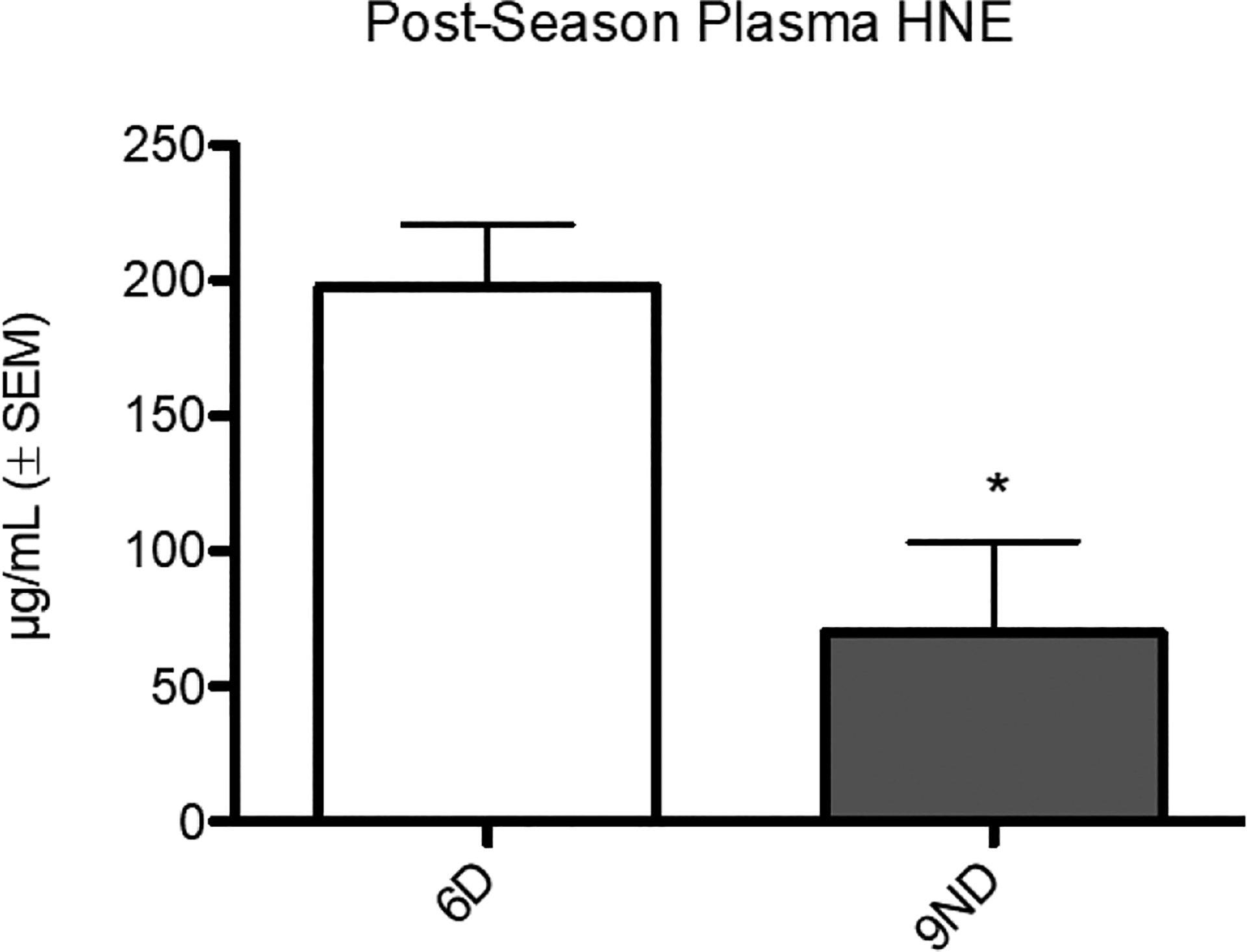
Post-Season Plasma HNE. In the post-season recovery condition, mean plasma HNE concentration was significantly higher (p < 0.05) for participants who reported at least one unexplainable drop in athletic performance lasting three weeks or longer during the competition season (6D) (197.5 μg/mL ± 22.79) than for participants who did not report such a drop (9ND) (69.80 μg/mL ± 33.59).

No significant difference in mean HNE concentration between mid-season (95.77 μg/mL ± 26.68) and post-season recovery (120.9 ± 27.22) was detected. Among the six participants who reported a drop in performance (6D), there was no significant difference between mid-season (143.1 ± 36.12) and post-season recovery (197.5 ± 22.79). There was no significant difference in concentrations between mid-season (64.19 ± 34.94) and post-season recovery (69.80 ± 33.59) for the nine participants who did not report a drop in performance (9ND). In the mid-season condition, there was no significant difference between 6D (127.1 ± 43.09) and 9ND (74.91 ± 34.15). In the post-season recovery condition, HNE concentration for 6D (197.5 ± 22.79) was significantly higher than 9ND (69.80 ± 33.59) (Figure 1).

Our analysis of NOX through nitrite ($\text{N}O_{2}^{-}$) and nitrate ($\text{N}O_{3}^{-}$) concentration revealed no significant difference in mean values between mid-season (21.35 μM ± 3.331) and post-season (20.59 ± 3.308). In the 6D group there was no significant difference between mid-season (23.44 ± 7.644) and post-season (24.61 ± 6.908). Data of 9ND showed no significant difference between mid-season (19.96 ± 2.723) and post-season (17.91 ± 3.130). In the mid-season, there was no significant difference between 6D (23.44 ±7.644) and 9ND (19.96 ± 2.723). In the post-season, there was no significant difference between 6D (24.61 ± 6.908) and 9ND (17.91 ± 3.3130).

Nitrotyrosine analysis detected no significant difference in mean concentrations between mid-season (131.3 nM ± 12.31) and post-season (151.6 ± 12.60). There were no significant differences in 6D between mid-season (152.4 ± 23.46) and post-season (151.9 ± 17.72) nor in 9ND between mid-season (118.2 ± 12.72) and post-season (151.4 ± 18.18). Nitrotyrosine concentrations between groups revealed no significant differences between 6D (152.4 ± 23.46) and 9ND (118.2 ± 12.72) in mid-season nor between 6D (151.6 ± 12.60) and 9ND (151.4 ± 18.18) in the post-season.

**Figure 2.**
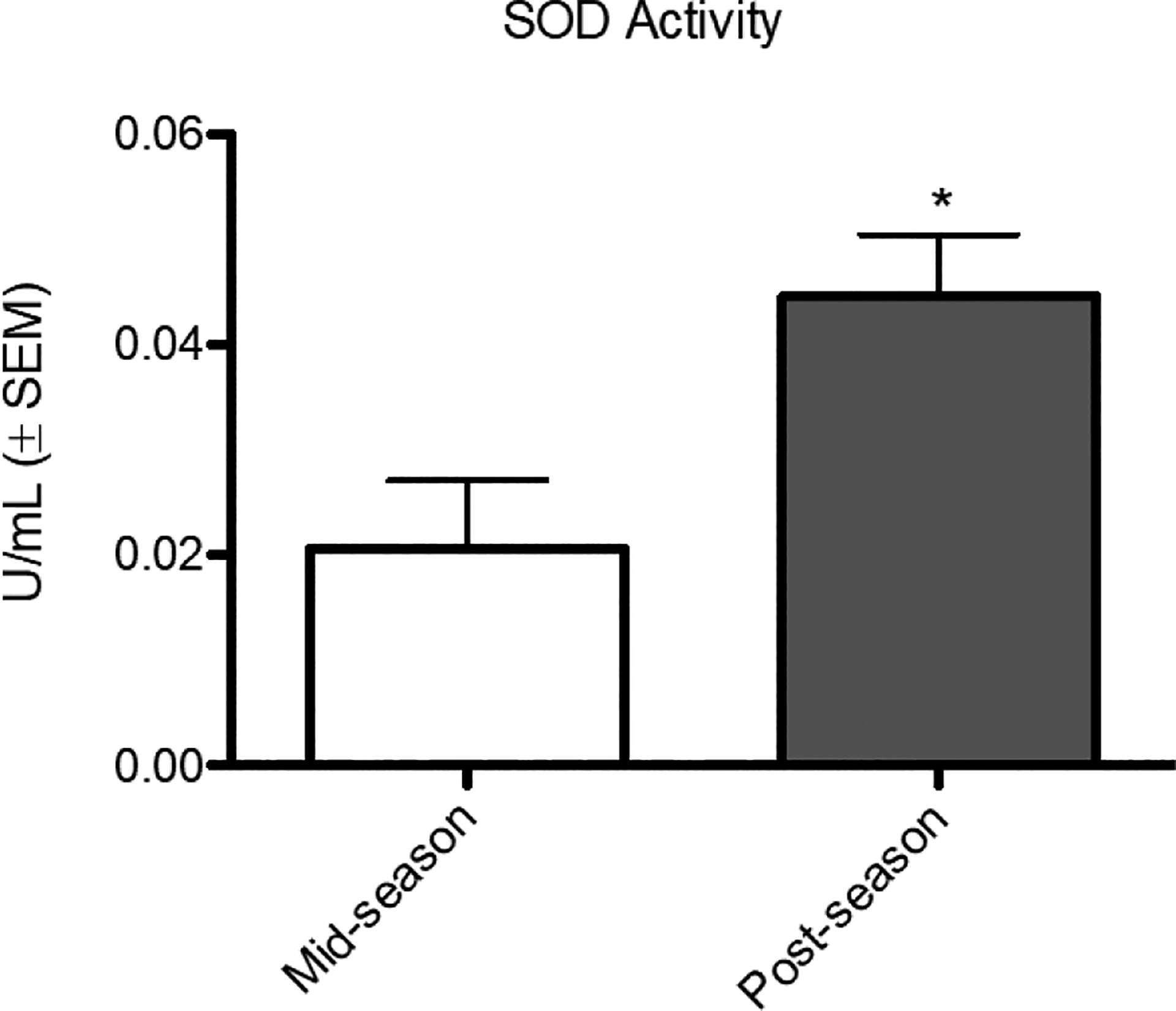
Comparison of superoxide dismutase mid-season and post-season. Participants displayed significantly higher (p < 0.05) SOD activity in the post-season recovery condition (0.02065 U/mL ± 0.006477) as compared to the mid-season condition (0.04459 U/mL ± 0.005860).

**Figure 3.**
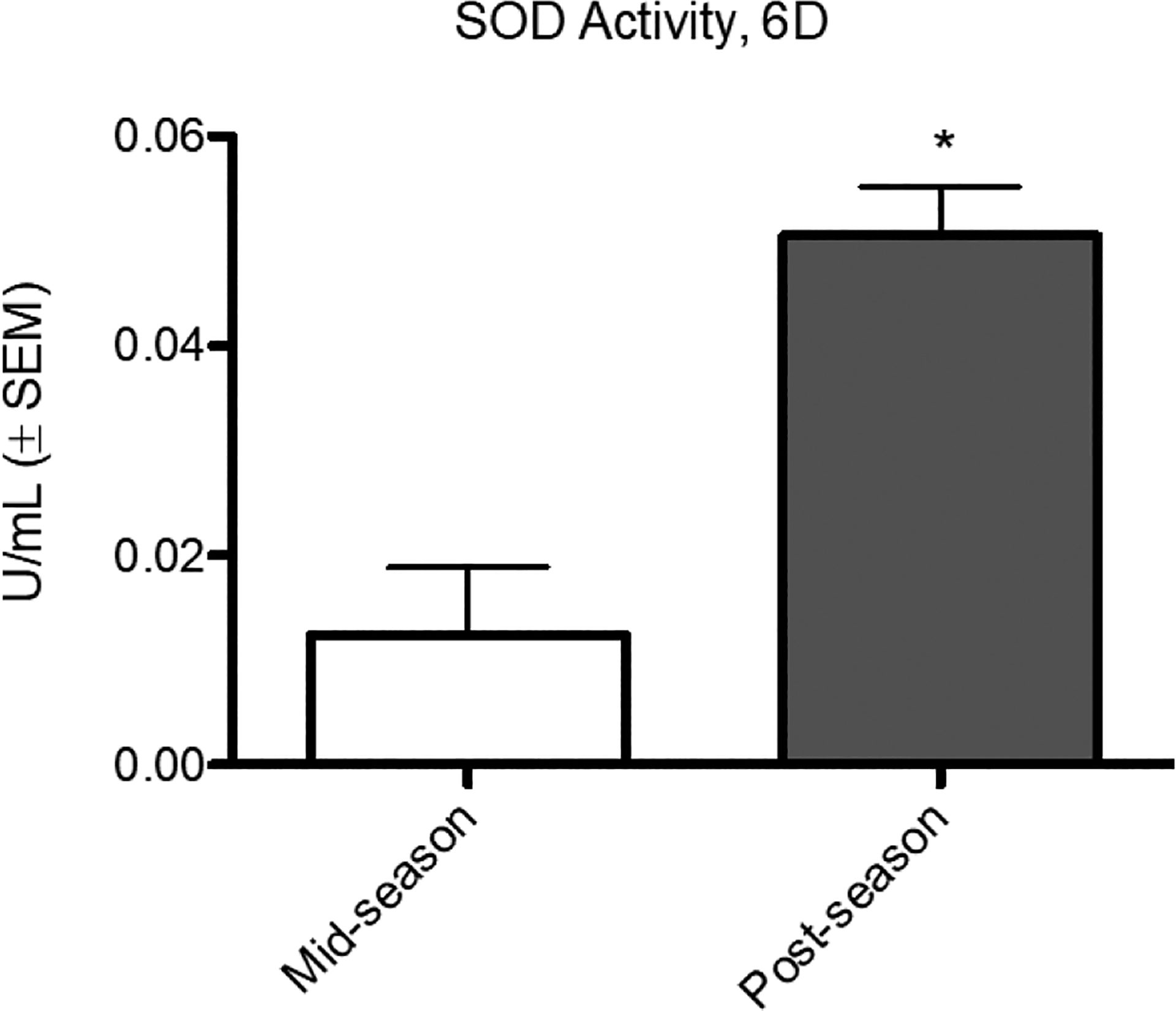
Comparison of superoxide dismutase of athletes reporting a drop in performance. Mean SOD activity for participants who reported at least one unexplainable drop in athletic performance lasting three weeks or longer during the competition season (6D) was significantly higher (p < 0.05) in the post-season condition (0.05048 U/mL ± 0.004688) than in the mid-season condition (0.01241 U/mL ± 0.006469).

Analysis of SOD revealed significantly lower activity in mid-season (0.02065 U/mL ± 0.006656) than post-season (0.04459 ± 0.005860) (Figure 2). Among 6D, there was significantly lower SOD activity in mid-season (0.01241 ± 0.006469) than post-season (0.05048 ± 0.004688) (Figure 3). Analysis of 9ND exhibited no significant difference between mid-season (0.02385 ± 0.009359) activity and post-season (0.04326 ± 0.009059). In mid-season, 6D (0.01241 ± 0.006469) and 9ND (0.02385 ± 0.009359) displayed no significant difference. In post-season, no significant difference was found between 6D (0.05048 ± 0.004688) and 9ND (0.04326 ± 0.009059).

## 4. Discussion

Production of reactive oxygen species (ROS) and reactive nitrogen species (RNS) is ubiquitous with human life [10] and is a nuanced and complex system. Numerous mechanisms, both endogenous and exogenous, contribute to ROS and RNS production. Primary sources of endogenous ROS include mitochondria [11], peroxisomes, endoplasmic reticulum, and phagocytic cells [12]. Exogenous sources include air pollution, radiation, alcohol, tobacco, heavy metals, transition metals, pesticides, industrial solvents, and certain drugs such as halothane (anesthetic) and acetaminophen (analgesic/fever reducer) [12]. Relative to exercise, mitochondrial respiration is the most important production mechanism for free radicals. Electrons “leak” from the electron transport chain via semiquinone anion, namely in complex I and complex III, and join molecular oxygen to form superoxide ion radical [12]. The non-enzymatic nature of ROS production through mitochondrial respiration means that at a higher metabolic rate more ROS is produced [12]. A “crucial balance” between ROS and RNS production and antioxidant defense may play an important role in endogenous disease prevention [10]. Oxidative stress from exercise and/or environmental toxins can cause a disruption in this crucial balance.

A healthy system will overcome oxidative stress through endogenous and exogenous antioxidants. A normal return to homeostasis typically occurs with isolated or short-term oxidative stress. For example, regular bouts of moderate exercise will initially increase ROS and RNS. Homeostasis will be achieved, however, if the body adjusts to greater demands on mitochondrial respiration. As such, the “crucial balance” will be reclaimed. However, if oxidative stress outstrips the body’s ability to adjust, by frequency and/or magnitude, disease may follow. In the case of endurance athletes, OTS may result [1]. ROS and RNS induced by oxidative stress have been linked to diabetes mellitus (DM), neurodegenerative diseases, cancer, cardiovascular diseases (CVD), cataract lenses, rheumatoid arthritis, and asthma [12]. Abnormal inflammation, a condition associated with a series of risk factors for metabolic diseases including DM, CVD, obesity [13], and rhabdomyolysis [14], is closely associated with oxidative stress and is a central pillar of the cytokine theory of OTS [1].

Lipid peroxidation is characterized by the oxidation of lipid molecules by free radicals. Polyunsaturated fatty acid residues of phospholipids in cellular lipid membranes are particularly vulnerable to peroxidation [12]. Through a cascade of events including the formation of a lipid radical (L^•^), then a peroxy radical (LOO^•^), the primary product of lipid peroxidation is formed, lipid hydroperoxides (LOOH). In addition to LOOH, several secondary products are formed including HNE, “…a major bioactive marker of lipid peroxidation…” [15]. Our data indicate that the group of participants who experienced at least one decline in athletic performance lasting at least three weeks during the competitive season had significantly higher mean HNE concentration than those who did not experience such a decline (Figure 1). Higher HNE concentration suggests a higher degree of lipid peroxidation and cellular damage in the 6D group than the 9ND group even after a one-month recovery period.

A vasodilator produced in epithelial cells, NOX reacts quickly with superoxide anion ($O_{2}^{\cdot -}$) to form a powerful and toxic oxidant, peroxynitrite ($\text{ONO}O^{-}$), continuing the oxidative cascade [16]. While NOX plays a critical role in mitochondrial respiration, and is an important signaling molecule, excess NOX can mediate oxidative damage and may downregulate energy production [17]. Due to the short half-life of NOX, quantification of NOX in samples is often performed by measuring its final products, $\text{N}O_{2}^{-}$ and $\text{N}O_{3}^{-}$. NOX is the only known biological molecule that, at high enough concentration, can outcompete endogenous SOD for $O_{2}^{\cdot -}$. We found no significant differences in $\text{N}O_{2}^{-}$ and $\text{N}O_{3}^{-}$ concentration in any of our comparisons suggesting that endogenous and exogenous $O_{2}^{\cdot -}$ scavengers may have outpaced $\text{ONO}O^{-}$ production.

Interaction between the amino acid tyrosine and $\text{ONO}O^{-}$ yields nitrotyrosine, a marker of nitrative stress and inflammation [18]. Protein-bound nitrotyrosine is present in a wide range of diseases with an inflammatory element including cardiovascular disease and diabetes [19]. Our data revealed no significant difference in mean concentration of nitrotyrosine between mid-season and post-season conditions for the entire group, 6D, and 9ND. No significant differences were found between 6D and 9ND in either condition. These findings suggest that oxidative protein damage may not have exceeded the ability to mitigate such damage.

$O_{2}^{\cdot -}$ is toxic and, unless an imbalance exists, is dismutated immediately by SOD to form hydrogen peroxide (H_2_O_2_) which, in turn, is reduced to H_2_O by catalases, glutathione peroxidases, and peroxiredoxins [20]. We tested for SOD activity as it “…is a first line of defense against toxicity of superoxide anion radicals” [20]. SOD from plasma was used as an oxidative stress biomarker although this may not actually reflect exact oxidation rates at the cellular level. Plasma levels are an accepted marker for oxidative stress analysis [9] [21]. While catalases, glutathione peroxidases, and peroxiredoxins are critical for clearance of radicals and a return to homeostasis, we chose to quantify SOD as its activity is an acceptable marker for oxidative stress response. Total SOD activity was measured which included for all three SOD metalloenzymes: cytosolic (SOD 1), mitochondrial (SOD 2), and extracellular (SOD 3). Mean SOD activity was significantly lower in mid-season as compared to post-season (Figure 2). For the group of participants who reported a drop in athletic performance, mean SOD activity was also significantly lower in mid-season as compared to post-season (Figure 3). For the overall group, SOD activity doubled in the post-season while for the 6D group the activity increased by a factor of four. The SOD activity for the entire group and for 6D contrasts with the mean SOD activity for 9ND which was not significantly different between mid-season and post-season. The 6D group was driving the mean SOD activity values and statistical significance for the entire group.

While it is clear that SOD activity for 6D was higher in the post-season recovery condition, there are several possible explanations. Higher SOD activity in 6D may indicate that a greater increase in $O_{2}^{\cdot -}$ production has occurred. Typically, increased production of $O_{2}^{\cdot -}$ takes place during exercise or periods of illness. All six participants of the 6D group reported experiencing at least one drop in performance lasting three weeks or longer during the competitive cross country ski season. It is unclear if the drop in performance is the only factor that accounts for the higher SOD activity in the post-season. Muscle damage and associated inflammation could potentially explain the increase in endogenous plasma antioxidant activity. Why SOD activity increased in the post-season and not in the middle of the competition season when stress levels, presumably, were higher would need explanation. What is clear from the SOD activity data is that members of the 6D group had something from which to recover. More extensive physical and biochemical examination would be beneficial during the season.

The SOD activity meshes somewhat with HNE data. In the post-season recovery condition, the 6D mean HNE concentration is over twice as high as the 9ND mean and significantly different (Figure 1). Higher SOD activity suggests that participants in the 6D group may have been experiencing higher lipid oxidation than participants in the 9ND group in the post-season recovery condition. The SOD activity discrepancy would support the notion that SOD activity was higher in 6D in the post-season due to oxidative stress. It is problematic, however, that there were no other significant differences in mean HNE concentrations in any of the other four comparisons. The sensitivity and relatively high level of standard error associated with each mean may explain at least part of this question. There are no extensive studies showing that the biomarkers actually correlate. It is likely that a larger sample size would clarify the seeming inconsistency; however, these larger studies present logistical challenges. Our study did not track diet nor environmental toxins entering the body through inhalation, ingestion, and absorption. A future study regarding OTS and oxidative stress should control for such variables as these have a direct bearing on overall oxidative stress. Additionally, this study did not account for individual variability in response to oxidative stress. Differences in methodology between studies addressing OTS in general, and OTS and oxidative stress in particular, need critical evaluation before drawing sweeping conclusions for the athletic community.

Our study identified six participants who displayed at least one athletic performance symptom associated with OTS. Samples from the six participants revealed higher SOD activity in the post season. The samples also displayed higher HNE concentration as compared to 9ND in the post-season, which is consistent with oxidative stress for the 6D group. This study provides additional support to the oxidative stress hypothesis of OTS. It also suggests that athletes may benefit from regular monitoring of markers of oxidative stress. A more robust sample size with a project design that includes quarterly sampling for an entire year should advance the understanding of the links between oxidative stress and athletic performance.

## Figures and Tables

**Table 1.** Participant characteristics.

	N	Age	Body fat (%)		Total MET^†^ (min/wk)	
			Mid-season	Post-season	Mid-season	Post-season
Total	15	20.4 ± 1.64	NA	NA	5335 ± 427	3185** ± 446
Male	8	20.6 ± 1.92	12.0 ± 0.387	11.9 ± 0.573	4903 ± 449	3447 ± 735
Female	7	20.1 ± 1.35	29.4 ± 2.22	28.7 ± 2.29	5830 ± 754	2886* ± 495

Age, percent body fat, and MET minutes/week for all participants, males, and females. Mean values ± SEM.

† Metabolic Equivalent Task.

** p < 0.01;

* p < 0.05.
